# Potential of laser‐induced fluorescence‐light detection and ranging for future stand‐off virus surveillance

**DOI:** 10.1111/1751-7915.13698

**Published:** 2020-11-26

**Authors:** Oloche Owoicho, Charles Ochieng’ Olwal, Osbourne Quaye

**Affiliations:** ^1^ West African Centre for Cell Biology of Infectious Pathogens (WACCBIP) University of Ghana Accra Ghana; ^2^ Department of Biochemistry, Cell and Molecular Biology College of Basic and Applied Sciences University of Ghana Accra Ghana

## Abstract

Viruses remain a significant public health concern worldwide. Recently, humanity has faced deadly viral infections, including Zika, Ebola and the current severe acute respiratory syndrome coronavirus 2 (SARS‐CoV‐2). The threat is associated with the ability of the viruses to mutate frequently and adapt to different hosts. Thus, there is the need for robust detection and classification of emerging virus strains to ensure that humanity is prepared in terms of vaccine and drug developments. A point or stand‐off biosensor that can detect and classify viruses from indoor and outdoor environments would be suited for viral surveillance. Light detection and ranging (LiDAR) is a facile and versatile tool that has been explored for stand‐off detection in different environments including atmospheric, oceans and forest sensing. Notably, laser‐induced fluorescence‐light detection and ranging (LIF‐LiDAR) has been used to identify MS2 bacteriophage on artificially contaminated surgical equipment or released amidst other primary biological aerosol particles in laboratory‐like close chamber. It has also been shown to distinguish between different picornaviruses. Currently, the potentials of the LIF‐LiDAR technology for real‐time stand‐off surveillance of pathogenic viruses in indoor and outdoor environments have not been assessed. Considering the increasing applications of LIF‐LiDAR for potential microbial pathogens detection and classification, and the need for more robust tools for viral surveillance at safe distance, we critically evaluate the prospects and challenges of LIF‐LiDAR technology for real‐time stand‐off detection and classification of potentially pathogenic viruses in various environments.

## Graphical abstract

Viruses remain a significant public health concern worldwide, and the threat is associated with the ability of the viruses to mutate frequently and adapt to different hosts. A point or stand‐off biosensor that can detect and classify viruses from indoor and outdoor environments would be suitable for viral surveillance. This review critically evaluates the prospects and challenges of LIF‐LiDAR technology for real‐time stand‐off detection and classification of potentially pathogenic viruses in various environments.

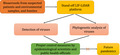

## Introduction

Microbial pathogens such as bacteria, viruses, fungi and parasites are a major public health concern. Of these, viruses have proven to be a great threat in recent times. Humanity has been faced with deadly viral infections including Zika virus, Ebolavirus and the ongoing severe acute respiratory syndrome coronavirus 2 (SARS‐CoV‐2) pandemic. The viral threat is attributed to their ability to mutate frequently and adapt to different hosts. To outcompete these viruses, there is the need for robust detection and surveillance of emerging strains. This will ensure that humanity is prepared in terms of vaccine and drug development. A promising approach in this respect is a point or stand‐off biosensor that can detect and classify viruses phylogenetically or based on their pathogenicity from indoor and outdoor environments. Stand‐off detection and identification systems are defined by their ability to detect and classify chemical, biological and explosive (CBE) hazards without contact with the hazardous material(s). The stand‐off detection range may be considered short (also referred to as non‐contact), medium or long, depending on the distance between the stand‐off detector and the material being targeted for detection. The stand‐off detection range has been variably defined. Even though some studies have presented < 10 m and > 10 m as short and long stand‐off range, respectively (Bogue, 2018), this review adopts the detection distances < 20 m, 20–100 m and > 100 m as short, medium and long stand‐off detection range, respectively, in consonance with many other reports (Sedlacek *et al*., 2002; Huestis *et al*., 2010; Babichenko *et al*., 2018; Fellner *et al*., 2020). Stand‐off detection methods are suitable for operations in high risk and harsh environments, as they could provide information about CBE hazards in real‐time from safe distances of several centimetres to up to a kilometre (Jonsson *et al*., 2009; Babichenko *et al*., 2018; Fellner *et al*., 2020). Based on these advantages, stand‐off detection will be an ideal approach for viruses, which can be highly contagious and dangerous.

Light detection and ranging (LiDAR) is a group of techniques that are based on the principle that when a pulsed laser (light amplification by stimulated emission of radiation) impinges on particles, the particles absorb and/or emit signals that can be characterized using a suitable detector (Buteau *et al*., 2008). The emitted signals could be a backward scattered or polarized light, or fluorescence (Yang *et al*., 2016). LiDAR is a facile and versatile tool that has been applied in remote environmental monitoring, including atmospheric, oceans and forest sensing (Eitel *et al*., 2016; Almeida *et al*., 2019). LiDAR has also become a powerful air‐ or space‐borne altimetric tool (Flood, 2001; Forfinski‐Sarkozi and Parrish, 2016). In addition, the LiDAR sensor is fast becoming a key component of autonomous vehicles, to provide information about the surroundings of the vehicles with high resolution (Royo and Ballesta‐Garcia, 2019; Tang *et al*., 2020).

Based on the nature of the scattered light, four basic types of LiDAR, including Rayleigh, Mie, Raman and fluorescence have been described (Veerabuthiran, 2003; Zhang *et al*., 2011). The laser‐induced optical behaviours have been exploited for the detection and characterization of biogenic materials both in laboratory and field conditions using LiDAR platforms. Raman scattering, for example, has been applied spectroscopically to detect and identify pathogenic bacteria in clinical samples (Ho *et al*., 2019) and determine antibiotics susceptibility and transcriptomic profile that reflect antibiotics resistance in bacteria (Germond *et al*., 2018). A combination of Rayleigh and Raman scattering effectively characterized extracellular vesicles (EVs) and lipoproteins, including differentiating tumour‐derived EVs from normal blood cells (Enciso‐Martinez *et al*., 2020a,b). Similarly, laser‐induced fluorescence (LIF) has been used for detection and/or identification of microorganisms and pollens (Pan *et al*., 2010, 2011; Babichenko *et al*., 2018; Swanson and Huffman, 2018).

Of the different types of LiDAR, fluorescence LiDAR, particularly the LIF‐LiDAR, has demonstrated high potentials for stand‐off detection and characterization of primary biological aerosol particles (PBAPs) (Buteau *et al*., 2008; Li *et al*., 2019). LIF‐LiDAR has been used to identify MS2 bacteriophage on artificially contaminated surgical equipment (Babichenko *et al*., 2018). LIF‐LiDAR has also been used to identify MS2 bacteriophage amidst other PBAPs in laboratory‐like close chamber and open field settings (Sivaprakasam *et al*., 2004; Baxter *et al*., 2007; Jonsson *et al*., 2009; Farsund *et al*., 2010) and thus highlighting the potentials of the technique for viral detection indoors or outdoors. In addition, LIF‐LiDAR was used to both detect and identify each virus from a pool of pure isolates of seven viruses of the Picornavirus family (Gabbarini *et al*., 2019). However, the potentials of the LIF‐LiDAR technology have not been assessed for real‐time stand‐off surveillance of pathogenic viruses in the environment. In view of the increasing applications of LIF‐LiDAR for potential pathogens detection, and the need for more robust tools for non‐contact viral surveillance at long range, this review critically evaluates the prospect and challenges of LIF‐LiDAR technology for stand‐off surveillance of potentially harmful viruses in in‐built or outdoor environments.

In this review, we critically provide an overview of the types of LiDAR, LIF‐LiDAR working principles, suitability and prospects of LIF‐LiDAR for virus surveillance, challenges of LIF‐LiDAR approach in virus surveillance and a perspective on overcoming the potential challenges.

## Types of LiDAR

**Fig. 1:**
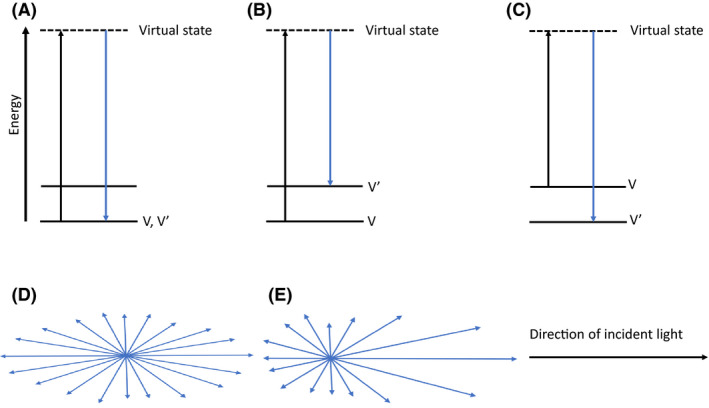
Schematic representations of Rayleigh, Mie and Raman scattering. A. Energy diagram of Rayleigh scattering. There is no change in frequency of the incident and scattered light (V = V’). B. Energy diagram of Stokes Raman scattering. There is a shift in frequency of the scattered light such that (V> V’). C. Energy diagram of anti‐Stokes Raman scattering. There is a shift in the frequency of the scattered light such that (V < V’). D. Rayleigh scattering. This occurs when the wavelength of the incident light is greater than the diameter of the impinged particle (e) Mie scattering. This occurs when the wavelength of the incident light is less than the diameter of the impinged particle. V = frequency of incident light. V’ = frequency of scattered light.

Based on the nature of the scattered light, many types of LiDAR, including Rayleigh, Mie, Raman and resonance fluorescence, have been described (Veerabuthiran, 2003; Zhang *et al*., 2011). Both Rayleigh and Mie scattering are elastic, meaning both incidence and scattered light have the same wavelength, and no energy is lost as the incidence light impinges on the particles (Veerabuthiran, 2003). For Rayleigh, the incident light has a much longer wavelength than the size of the impinged particle, while Mie scattering occurs when the particle has much larger size than the wavelength of the impinging light (Veerabuthiran, 2003). Unlike Rayleigh and Mie scattering, Raman scattering is inelastic and involves energy (or wavelength) shift, based on the molecular nature of the particles impinged by the light; the Raman energy shift is unique to different molecules and could be used to characterize different particles (Turner *et al*., 2016). Resonance fluorescence scattering is also inelastic and occurs when the wavelength of the incidence light corresponds to the absorption line of the atoms, ions or molecules, such that the atoms are excited to a higher energy level by the incoming light and re‐emit photons of the same or longer wavelength than the incident light (Abo, 2005; Gardner and Collins, 2015). Although elastic scatterings could be used to map clouds and detect PBAPs such as pollens, bacterial and fungal cells, or spores at low concentrations and long range, those types of LiDAR have poor resolution for particles of different composition but similar sizes. A detailed characterization of biological particles at long range will therefore require inelastic scattering (Simard *et al*., 2004; Buteau *et al*., 2006). Figure 1 summarizes the differences between Rayleigh, Mie and Raman scattering.

## LIF‐LiDAR: Working principles and its suitability for viral surveillance

The detailed working principle of LIF‐LiDAR has been well described in various reports (Joshi *et al*., 2013; Wojtanowski *et al*., 2015; Yang *et al*., 2016). Briefly, a LIF‐LiDAR has a source which emits laser of desired excitation wavelength to be directed to a target such as bioaerosols, and a receiving system which collects the echoed signals (such as a scattered light and fluorescence) from the laser‐interrogated target. Typically, a receiving system is comprised of a telescope which collects the scattered light, a dichroic mirror which separates the light into spectra based on frequencies, an array of photomultiplier tubes which receive high frequency light for signal amplification, and a spectrograph which receives and directs the lower frequency (longer wavelength) light to gated intensified charge coupled device (ICCD) camera (Joshi *et al*., 2013). In addition, a LIF‐LiDAR system has a data acquisition platform, which displays the amplified signals in a visible/readable format.

Microorganisms contain biomolecules in various subcellular structures and the biomolecules possess intrinsic fluorescence ability (Yang *et al*., 2016). Hence, when microorganisms are impinged with light of an appropriate wavelength, they emit fluorescence of maximal intensity (Yang *et al*., 2016). The fluorescence excitation and emission spectra are a function of the nature of fluorophores (the fluorogenic biomolecules) in the biomolecular architecture of the microorganisms and are discriminatory enough for identification and classification of the microorganisms (Buteau *et al*., 2006; Joshi *et al*., 2013). Viruses vary in their structures, even though they are basically comprised of nucleic acid (DNA or RNA) encased in a protein capsid (Louten, 2016) and some viruses, in addition to this basic structure, also possess enzymes and lipid‐derived envelopes (Mesters *et al*., 2006). Altogether, viruses possess different fluorophores that can be interrogated by light/laser to identify and/or classify them.

## Prospects of LIF‐LiDAR for viral detection and classification

The fluorogenic ability of viruses has been well‐established. Sivaprakasam and colleagues reported fluorescence intensities of 16 bioaerosols, including MS2 bacteriophage, at 350, 450 and 550 nm spectra bands when the bioaerosols were excited with 266 and 355 nm laser pulses at 400 nanosecond intervals (Sivaprakasam *et al*., 2004). Notably, the highest fluorescence intensity was obtained for the bacteriophage at the 350 spectra band and 266 nm excitation wavelength. At the two excitation wavelengths, the spectral signature of MS2 bacteriophage was discriminatory against 15 aerosol particles, including vegetative bacteria, spores and proteins. In contrast, a small scale LIF‐LiDAR platform built to remotely detect biological weapons using a single excitation wavelength of 266 nm showed a significant overlap (14.8%) between MS2 bacteriophage and dirty diesel exhaust fluorescence spectra (Baxter *et al*., 2007). This discrepancy highlights the potentials of multiwavelength excitation to increase the discriminatory power of LIF‐LiDAR for virus detection and identification in aerosols.

Field trials of two LiDAR systems, namely from the Norwegian Defense Research Establishment (FFI) and Swedish Defense Research Agency (FOI), have been reported for simulated detection of different bacterial species, ovalbumin and MS2 bacteriophage, in a plume of expected atmospheric interferents, such as pollens, oil fog, smoke and diesel fumes have been reported (Jonsson *et al*., 2009). Using 355 nm excitation wavelength pulsed at 5 ns and 10 Hz pulse repetition frequency (PRF), fluorescence spectra with peak within the 450 nm band was acquired for the MS2 bacteriophage released at a concentration of 3 × 10^9^ – 1.5 × 10^11^ plaque‐forming units per millilitre (PFU/ml), 230 m – 1 km away from the LiDAR systems and 100–200 m upwind. A comparison of 355 and 294 nm excitation wavelengths for stand‐off detection of bioaerosols containing bacterium, MS2 bacteriophage and ovalbumin released in a semi‐closed chamber that was 210 m away from the LIF‐LiDAR platform indicated a better spectral resolution with the 294 than 355 nm (Farsund *et al*., 2012). Farsund et al. also observed highest fluorescence intensity for MS2 bacteriophage within the 350 nm spectral band when the virus was excited with the 294 nm wavelength. In another study, a LiDAR platform named BC‐Sense device obtained a unique spectral signature of 430 nm maximal fluorescence for MS2 bacteriophage when the virus was excited with a 248 nm laser of PRF of 10 Hz (Babichenko *et al*., 2018). The spectral profile of BC‐Sense device was discriminatory enough to identify MS2 bacteriophage on several surfaces that were co‐contaminated with bacteria and fungi and placed about 20 m away from the LIF‐LiDAR with a limit of detection of 9.5 × 10^4^ PFU/cm^2^. However, this limit of detection is high and may not be suitable for stand‐off virus surveillance.

Autofluorescence of F2 bacteriophage has been observed to depend almost entirely on amino acid contents (particularly tryptophan and tyrosine) of its nucleocapsid rather than its RNA genome; when F2 bacteriophage was excited with a UV light of 265–295 nm, it emitted fluorescence spectra within 320 nm band (Kitchell *et al*., 1977). A latter study using two bacteriophages, Ø6 and Ø12, and their bacterial hosts showed that the fluorescence signature of tryptophan varies with different protein environments and hence tryptophan fluorescence profile of the two phages differs from their *Pseudomonad* hosts (Alimova *et al*., 2004). These findings suggest that, with appropriate laser excitation wavelengths, viruses could be discriminated from bacteria and other biogenic materials.

A study which investigated spectrally resolved fluorescence cross sections of bioaerosols at 266, 273, 280, 365 and 405 nm excitation wavelengths reported excitation‐emission spectra of Venezuelan equine encephalitis virus (VEEV), as well as MS2 bacteriophage and several vegetative bacteria and spores. VEEV and MS2 bacteriophage strongly emitted fluorescence within 280–400 nm spectra band, with peak fluorescence occurring at 310–320 nm, when the viruses were interrogated with 266, 273, and 280 nm wavelengths (Pan *et al*., 2014). Interestingly, Pan and colleagues also observed dissimilar excitation‐emission signatures between VEEV and MS2 bacteriophage, with the former showing strong fluorescence only within the 280–400 nm band, while the latter had strong fluorescence within 280–400 nm and 400–600 nm bands. VEEV and MS2 bacteriophage belong to two different families of viruses, *Togaviridae* and *Leviviridae*, respectively, and suggest that viruses belonging to different families may have different excitation‐emission spectra signature that could be explored for LIF‐based viral classification.

A LIF platform was used to identify seven viruses that belong to the Picornavirus family. When the viral samples were interrogated with a 266 nm laser of 10 kHz PRF in the LiDAR set up, the viruses emitted fluorescence spectra between 350 and 700 nm. These spectra signatures were unique to the viruses except for two serotypes—Coxsackie A7 and A9, which had poor separation based on principal component analysis (PCA) (Gabbarini *et al*., 2019). However, further analysis showed that support vector machine (SVM), a supervised machine learning algorithm developed for classification and regression analysis, especially using complex data (Bhavsar and Panchal, 2012; Pisner and Schnyer, 2020) and neural network algorithm, perfectly classified the seven viruses investigated. Further, the limit of detection of LIF platform was 2 × 10^4^ – 2 × 10^5^ tissue culture 50% infectious dose (TCID_50_)/ml for hepatitis A, although the limit of detection was not determined for the other six viruses.

Collectively, the studies highlighted above indicate that viruses have excitation–emission spectral profile that could be explored to differentiate them from one another, from other microbial agents and from environmental interferents such as pollens and hydrocarbon effluents. However, despite these promising findings, the suitability of LIF‐LIDAR for stand‐off viral surveillance in real‐life to warrant field deployment of the technique has not been established.

## Challenges to LIF‐LiDAR approach in viral surveillance

First, most studies reporting on viral fluorescence spectra used purified viral isolates and hence the results obtained may not reflect a real‐life environment where viruses exist amidst plethora of chemical and biological interferents (Pan, 2015). Even though environmental interference was simulated in some of the studies in the previous section, the spectrum of the interfering simulants used was too narrow to capture the varieties of PBAPs and other aerosol components in the atmosphere.

Second, the concentration of viruses in the atmosphere, indoor air or surfaces, may be less than those detectable by the LIF platforms tested. Although data on viral concentration in indoor and outdoor air are scant, total virus‐like particles (VLPs) of 1 × 10^5^/m^3^ and 2.6 × 10^5^/m^3^ have been reported for indoor and outdoor air, respectively (Prussin *et al*., 2015). In addition, a metagenomics‐based study in Korea indicated that the VLPs concentration of near surface atmosphere (about 1 m above the ground level) varies seasonally from 1.7 × 10^6^ to 4.0 × 10^7^ VLP/m^3^ with winter and spring having the highest and lowest VLP concentrations, respectively (Whon *et al*., 2012). The total VLPs in outdoor environments that were reported by Whon *et al*. and Prussin *et al*. were lower than the viral concentration that was measured in the Jonsson *et al*., 2009 study (3 × 10^9^ – 1.5 × 10^11^ PFU/ml). Although the reported total VLP concentrations are comparable to the 10^4^ PFU/cm^2^ and 2 × 10^4^ – 2 × 10^5^ TCID_50_/ml limits of viral detection of the LIF platforms used by Babichenko et al. and Gabbarini et al., respectively (Babichenko *et al*., 2018; Gabbarini *et al*., 2019), the limits of detection of the LIF platforms are likely less than the atmospheric viral concentrations when individual viruses are considered rather than total VLP.

Third, bioaerosol surveillance using LIF‐LiDAR requires the use of high‐energy pulsed excitation laser (Baxter *et al*., 2007; Farsund *et al*., 2012) and this raises genuine biosafety concerns, especially that a high‐energy laser could cause irreversible damage to the eyes (Sayed, 2014; Birtel *et al*., 2017). Although some researchers have tried to reduce the high‐energy laser to eye‐safe energy level by enlarging the beam radius of the laser to 10 mm (Duschek *et al*., 2017), biosafety is still not guaranteed.

Fourth, a key advantage of the LIF‐LiDAR is the potential for a long‐range detection of harmful bioaerosols, especially those classified as biological weapons. However, choosing the right excitation wavelength for stand‐off interrogation of viruses in the atmosphere faces some difficulties. For instance, whereas studies have indicated that shorter excitation wavelengths (248–294 nm) elicit more intense and distinct fluorescence spectra for viruses than longer excitation wavelengths such as 355 nm (Sivaprakasam *et al*., 2004; Farsund *et al*., 2012; Babichenko *et al*., 2018), the shorter wavelengths are more easily attenuated in the atmosphere than the longer wavelengths. Hence, choosing the appropriate excitation wavelength may be difficult.

The fifth hurdle is about the cost of LIF‐LiDAR platforms, which is due to various factors; it is expected that the cost will be directly proportional to the stand‐off distance, and the area to be monitored among other factors. The average cost of UV‐LIF bioaerosol sensors runs into hundreds of thousands of US Dollars (Swanson and Huffman, 2018), which is prohibitive for their commercialization and routine application, especially in poor‐resourced countries, which coincidentally have higher burden of infectious pathogens, including viruses (Fenollar and Mediannikov, 2018).

## LIF‐LiDAR for viral surveillance: What will it take?

### Excitation‐emission spectra of viruses are needed

Although extensive characterization of laser excitation‐emission spectra of bacteria, fungi and pollens have been undertaken (O’Connor *et al*., 2011; Pöhlker *et al*., 2011; Dartnell *et al*., 2013), similar studies on viruses are lacking. Hence, to adapt LIF‐LiDAR for stand‐off viral surveillance, studies are needed to determine the optimum excitation–emission spectra of viruses that are potentially highly pathogenic to humans. This baseline data are required for building databases that are needed for real‐time detection, classification and identification of viruses. Such studies should consider irradiating different classes of virus with a range of UV‐laser excitation wavelengths to determine laser specifications that would yield maximum intensity and most distinct signature spectra for individual viruses and classes of viruses.

### A multiwavelength excitation approach is required

A key requirement for viral surveillance using LIF‐LiDAR is the ability to discriminate viruses from other microbes or materials. Viruses differ from microorganisms, in terms of biomolecular composition, in many respects. Notably, reduced nicotinamide adenine dinucleotide (NADH), a redox carrier and co‐enzyme in energy metabolic pathways in viable cells, and other co‐enzymes (e.g. riboflavin–vitamin B_2_) and vitamins which are fluorogenic, are absent in viruses. Interestingly, NADH is the most dominant fluorogenic co‐enzyme in microbial cells, with well‐characterized fluorescence excitation–emission spectra (Pöhlker *et al*., 2011). Direct excitation wavelength of NADH ranges from 340 to 370 nm while its emission wavelength is 440–470 nm (Kaye *et al*., 2005; Pöhlker *et al*., 2011; Babichenko *et al*., 2018; Fennelly *et al*., 2018). Reported excitation and emission wavelengths for viruses are mostly in the range of 248–295 nm and 310–450 nm, respectively (Sivaprakasam *et al*., 2004; Farsund *et al*., 2012; Babichenko *et al*., 2018; Gabbarini *et al*., 2019). Hence, probing bioaerosol clouds with excitations of 340–370 nm and 248–295 nm wavelengths may yield spectra features to discriminate viruses from viable bacteria and fungi. Similarly, non‐viable bacterial and fungal cells, and spores and pollens, which may lack NADH, equally possess fluorogenic biopolymers that could discriminate them from viruses (Pöhlker *et al*., 2011). Bacteria, fungi and pollens possess cellulose and sporopollenin, respectively, and in their cell walls, and fungi also have chitin as one of their cell wall components. These biopolymers are all absent in viruses. Interrogating a bioaerosol particle with carefully selected multiple wavelengths could discriminate viral from non‐viral particles based on their fluorescence spectra signatures.

### Size differences could enhance viral discrimination

Viruses could also be discriminated from other PBAPs based on their differences in size. Apart from members of the *Poxviridae* which are up to 300 nm in diameter, human pathogenic viruses are generally small, often less than 200 nm in diameter (Gelderblom, 1996). Bacteria and fungi, on the other hand, are generally larger than viruses. With the size differences, an excitation wavelength, which is smaller than microbial cells but larger than viral particles, will elicit Rayleigh and Mie scattering upon impinging a virus and bacterium or a fungus, respectively. It also suggests that Rayleigh scattering could be used to locate viruses among bacteria and fungi using < 300 nm excitation wavelengths.

### Discrimination of viruses by Rayleigh‐LIF‐LiDAR hyphenation and time‐resolved fluorescence

The differential capacity of Rayleigh scattering is enhanced in a system combining Rayleigh with an inelastic scattering (Enciso‐Martinez *et al*., 2020a,b). Hence, a LiDAR system for viral detection and identification in real‐life environment will potentially use a multiwavelength excitation approach to probe bioaerosol clouds to acquire Rayleigh scattering information which will help to locate the viruses and LIF spectra data for virus classification/identification. In addition, the specificity of the Rayleigh‐LIF‐LiDAR platform could be enhanced by using multiple channels of resolution for acquiring the LIF spectra (Kaye *et al*., 2005; Ruske *et al*., 2017). The specificity of the hyphenated LiDAR platform could further be enhanced by acquiring data on fluorescence lifetimes (time‐resolved LIF data) of the viruses and other aerosol particles, as was recently demonstrated for detection, classification and identification of some CBE agents (Fellner *et al*., 2020).

The time‐resolved LIF is based on observations that the fluorescence decay time depends on the immediate environment of the fluorophores. Some of the variables that may influence fluorescence lifetimes include the concentration of ions in the environment and the presence of neighbouring fluorophores (Meier *et al*., 2010). Since viruses differ from one another and from other PBAPs by amino acid composition, and lack co‐enzymes, such as NADH and riboflavin which are present in viable cells, the microenvironment, and by extension, the fluorescence decay time of fluorophores is expected to differ significantly among viruses and from other fluorogenic materials. This suggests that a combination of Rayleigh, LIF and time‐resolved LIF‐LiDAR will be a potentially robust platform for detection and specific identification of viruses in real‐life environments where interferents which possess overlapping fluorescence spectra may be present. However, for this to be achieved, studies are needed to generate reference data regarding the fluorescence lifetimes of viruses, bacteria, fungi, spores, pollens and other potential interferents in the atmosphere.

### Machine learning algorithms are required

Considering that a feasible LiDAR platform for viral surveillance will likely acquire both elastic and inelastic radiation data that involves multiple spectra features, analysis of the acquired data may be very challenging. The primary focus of the data analysis will be to determine whether an interrogated PBAP is a virus, what virus it is, and if it poses a threat or not. Machine learning algorithms (MLA) with high predictive power and trained with a robust database of viral spectra features and fluorescence lifetimes will be required to build an online virus classifying platform for real‐time viral surveillance. A decision tree (implemented in the online virus classifying platform) could be grown from the size, spectra, fluorescence lifetimes datasets and lists of potentially harmful viral agents to indicate if a virus identified poses a threat (positive alarm) or not (negative alarm), following a model that has previously been described (Fischbach *et al*., 2015).

Several MLAs, such as support vector machine, principal component analysis, decision tree, neural network and wavelet transform, have been used for extraction of relevant spectra features and reduction of high‐dimensional spectra data to lower dimensions and thereby reducing computational complexity and increasing classification accuracy (Sobolev and Babichenko, 2013; Gabbarini *et al*., 2019). Genetic algorithm (an evolutionary algorithm), which has higher classification accuracy than SVM, principal component analysis, Fisher’s linear discriminant and forward feature selection, has also been reported (Nyhavn *et al*., 2011). Future studies on viral surveillance using LiDAR could explore the appropriate algorithms, as well as consider other algorithms such as the t‐Distributed Stochastic Neighbour embedding, which is suitable for both linear and non‐linear data, for high‐dimensional spectra data reduction.

### Cost reduction and biosafety concern

There is a high potential for miniaturization and significant reduction of the cost of LIF‐LiDAR platforms for bioaerosol agents’ surveillance. Although early LIF platforms that have been built for bioaerosol surveillance require high end instrumentation and therefore make them quite expensive for widespread use, recent LIF platforms have used innovative instrumentation such as the replacement of Q‐switch solid‐state lasers with UV light‐emitting laser diodes (LED) as an effective and inexpensive laser excitation source (Zhang *et al*., 2013; Swanson and Huffman, 2018).

Biosafety concern should be thoroughly addressed before considering LIF‐LiDAR for viral surveillance, especially in residential or other places where people are likely going to be exposed to the high‐energy laser. Although the laser safety concern cannot be completely addressed presently, keeping the excitation wavelength below 400 nm, which is considered ‘eye‐safe’ (Franks, 1991) should be considered. In addition, standard safety precautions for use of laser, including laser‐safe eye goggle should be mandatory (Smalley, 2011).

## Conclusion

Viral infections have caused unprecedented hardship to humanity, partly due to the ability of viruses to mutate fast and adapt to new hosts. To manage and avert viral infections that evolve into outbreaks like the current COVID‐19 pandemic, robust viral surveillance and monitoring of emerging viruses is imperative. Here, we have assessed the potential of LIF‐LiDAR approach for detection of viruses before, during and beyond pandemics, by looking at the prospects, challenges and perspectives of the approach that may be associated with stand‐off viral surveillance. Taking together all the factors that have been addressed in this review, we conclude that substantial effort is still required to achieve a long‐distance viral surveillance in the atmospheric environment using the LIF‐LiDAR approach. However, the platform may have successful short stand‐off range applications in food and medical virology. For instance, LIF‐LiDAR is amenable to virus surveillance on fomites such as medical equipment, medical laboratory workbench, theatre tables and conveyor belts in food industries, among others.

## Conflict of interest

The authors have declared that no competing interests exist.

## Author contributions

OO and COO conceived and prepared the first draft of the manuscript. OQ critically reviewed the draft. All the authors approved the final version of the manuscript.
